# The decision-making process in the choice of VBAC: Facilitators and barriers from women’s perception, a systematic review

**DOI:** 10.18332/ejm/205874

**Published:** 2025-06-30

**Authors:** Greta Cosmai, Maria Biondini, Maria Panzeri, Marzia Serafini, Laura Lambicchi, Anna Locatelli, Antonella Nespoli, Simona Fumagalli

**Affiliations:** 1 University of Milano Bicocca, Milano, Italy; 2 Department of Obstetrics and Gynecology, Fondazione IRCCS San Gerardo dei Tintori, Monza, Italy

**Keywords:** VBAC, TOLAC, cesarian section, decision-making, midwives, woman’s experience, woman’s perception

## Abstract

**INTRODUCTION:**

Vaginal birth after cesarean (VBAC) is associated with a lower risk of maternal morbidity, fewer complications in future pregnancies, and a reduced overall cesarean section (C-section) rate at the population level. Despite these benefits, a woman’s decision for VBAC is shaped by multiple factors. This review aims to identify elements perceived by women as influential in the VBAC decision-making process.

**METHODS:**

We conducted a systematic review between 1 June and 12 July 2024, using PubMed, CINAHL, Embase and PsycINFO. We included English-language studies (2014–2024) on women eligible for VBAC with ≥1 previous C-sections. Study quality was appraised using CASP. Findings were narratively and thematically synthesized.

**RESULTS:**

Twenty-one studies met the inclusion criteria. Fifteen recurring factors were identified. ‘Facilitators’ of VBAC included: mother–newborn bonding; support from partner and family; desire for vaginal birth; previous VBAC experience; shorter postpartum recovery; partner and family support; social support from other women; healthcare professionals’ attitudes; communication and respectful maternity care; and counseling. ‘Barriers’ included: anxiety and fear of the unknown; healthcare professional’s misinformation and attitudes; coercive counseling; pain related to labor; loss of control; and perceived risk to mother or newborn.

**CONCLUSIONS:**

VBAC decision-making is influenced by past birth experiences, perceived support, and current concerns. Healthcare professionals’ attitudes and high-quality counseling are key to informed, unbiased choices. Continuity of care, midwifery care and education can empower women and reduce unnecessary C-sections. However, as most studies are from high-income, English-speaking countries, findings may not generalize globally.

## INTRODUCTION

Vaginal birth after a previous cesarean section (VBAC) represents a significant challenge for both women and healthcare professionals. Historically, it was considered a high-risk option due to concerns about uterine rupture, a belief deeply rooted in past obstetric practice. However, current evidence demonstrates that, for most women with a previous C-section, VBAC is a safe and beneficial alternative.

VBAC is now recognized as a reasonable and evidence-based choice for many women with a history of cesarean delivery. Those who choose VBAC can avoid surgery-related risks such as hemorrhage, thromboembolism, infection and prolonged hospitalisation1. Additionally, they often report greater maternal satisfaction and a sense of empowerment from achieving a desired vaginal birth^2^. For women planning future pregnancies, VBAC is also associated with a reduced risk of maternal complications linked to multiple cesarean deliveries, including hysterectomy, bowel or bladder injury, transfusion, infection, abdominal adhesions and abnormal placentation^1^. In terms of neonatal outcomes, VBAC carries a slightly increased risk of perinatal mortality and hypoxic-ischemic encephalopathy – mostly related to uterine rupture – when compared to elective repeat C-section. Nonetheless, the absolute risk remains low and is comparable to that observed in nulliparous women admitted in labor^2^. Moreover, VBAC is associated with higher breastfeeding rates and lower respiratory morbidity. Promoting VBAC should be considered a public health priority to reduce overall cesarean section rates particularly given the high proportion of repeat C-sections performed without other indications^1^.

Public health strategies should focus on promoting clinical appropriateness, strengthening the role of healthcare professionals in safeguarding maternal and neonatal wellbeing and supporting women’s active participation in birth-related decisions-making^3^.

Counseling women with a previous C-section about their birth options remains a key challenge. Although an increasing number of women face the choice between an elective repeat cesarean and a trial of labor, limited research has explored the most effective ways to support this decision-making process^4^.

A woman’s choice regarding the mode of birth after a C-section is shaped by a complex interplay of psychological, emotional, physical, and social factors. These may include their overall health and well-being, as well as that of their babies^5^, the quality of perinatal counseling received, perceived risks, and the support offered by healthcare professionals. Previous birth experience, particularly those involving feelings of loss of control, inadequate support, prolonged recovery, or unexpected surgical complications, can strongly influence maternal preferences. Women who perceive their past C-section as traumatic or unsatisfactory are more likely to view VBAC as an opportunity to regain control over their birth experience. In contrast, those with a positive cesarean experience or who fear the unpredictability of labor may prefer a planned repeat C-section^6-12^.

Maternal perceptions have been identified as a key factor in the decision-making process regarding the mode of birth after a C-section. However, to our knowledge, the literature lacks a comprehensive synthesis of the elements that women perceive as most influential in this process. Such a synthesis could support healthcare professionals in providing personalized, women-centered care during the antenatal period.

To address the gap in the literature regarding the factors that influence women’s choice about birth mode after a C-section, this systematic review aims to identify which elements are perceived by women as influential in the decision-making process related to VBAC.

## METHODS

### Study design

A systematic review was conducted between 1 June and 12 July 2024, using four major databases: PubMed, CINAHL, Embase and PsycINFO. To ensure the inclusion of the most recent literature, a final search was performed in October 2024 to identify any newly published studies relevant to the topic.

### Search strategy

The search strategy was developed using a combination of Medical Subject Headings (MeSH) and free-text terms related to vaginal birth after cesarean. Keywords included: ‘vaginal delivery after cesarean*’, ‘VBAC’, ‘experience*’, ‘life experience’, ‘birthing experience*’, ‘decision’, ‘experience*’, ‘perception*’, ‘attitude*’, ‘sight*’, and ‘factor*’. Boolean operators (AND, OR) were applied to systematically combine terms and maximize the retrieval of relevant studies.

### Inclusion criteria

We applied the following inclusion criteria: 1) studies published in English between 1 January 2014 and 12 July 2024; 2) peer-reviewed articles; 3) primary research or literature reviews; 4) studies involving women with ≥1 previous C-sections who were eligible for VBAC; and 5) studies exploring women’s experiences, perceptions, or decision-making processes regarding VBAC. Grey literature, non-peer-reviewed articles, and non-English publications were excluded.

### Selection process

**Figure 1:**
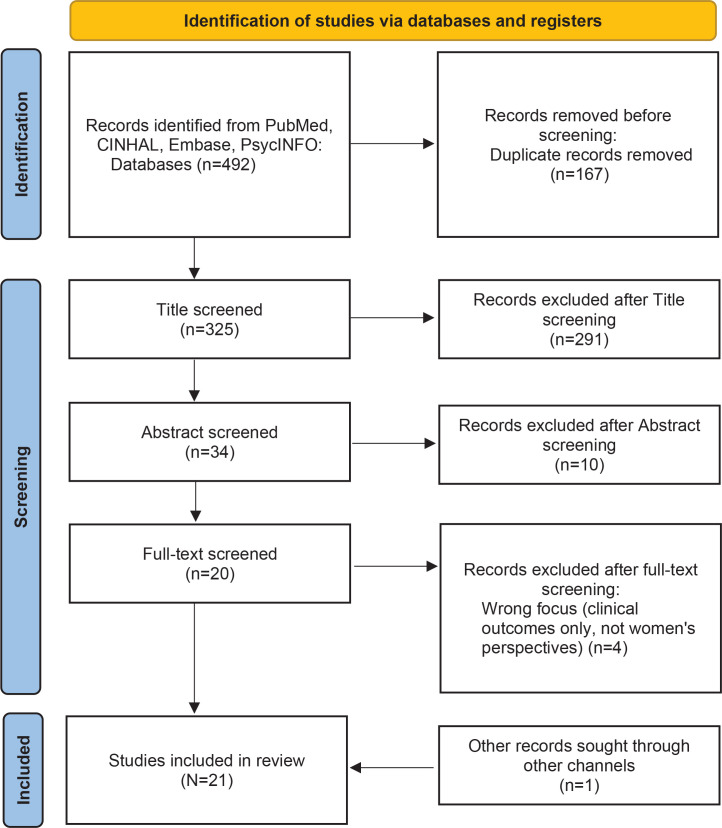
PRISMA flow diagram

The PRISMA flow diagram (Figure 1) describes the selection process, including identification, screening, eligibility assessment, and final inclusion. Two independent reviewers (GC and MB) assessed all identified studies for relevance, first by screening titles and abstracts, followed by fulltext evaluation based on predefined eligibility criteria. All references were stored in a shared database and duplicates were removed prior to full-text screening. Disagreements were resolved through discussion, with a third reviewer (SF) acting as arbitrator when necessary.

### Study quality

Study quality was assessed independently by GC and MB using the Critical Appraisal Skills Program^13^. The use of this tool ensured a rigorous evaluation of methodological quality, reducing the risk bias and systematic errors^14^. However, a formal certainty of evidence assessment was not conducted, as the review employed a narrative synthesis approach aimed at exploring thematic patterns rather than estimating quantitative effect sizes. This decision is consistent with current methodological guidance for qualitative evidence syntheses. The results were reported in accordance with the Preferred Reporting Items for Systematic Reviews and Meta-Analyses (PRISMA) guidelines^15^.

### Data items

Data extraction was conducted independently by GC and MB under the supervision of the third reviewer (SF). For each included study, the following variables were extracted: study design, country and setting, obstetric characteristics, methodology, and key findings related to women’s experiences, perceptions, or decision-making about VBAC. These data are summarized in Table 1. No major assumptions or simplifications were applied during the extraction process; any unclear elements were discussed and resolved through consensus among the reviewers.

**Table 1:** Characteristics of studies involved in the systematic review

*Authors Year Country*	*Title*	*Participants*	*Study design*	*Key findings*
Akgün and Boz^29^ 2019 Türkiye	*Women’s decision-making processes and experiences of vaginal birth after caesarean birth: A phenomenological study*	12 women with a successful VBAC within two years	Qualitative phenomeno-logical	Women have defined vaginal delivery after a C-section an important aspect of their femininity and sense of motherhood, life experience; a way to remove traumatic experiences of C-section delivery; a mode of birth that promotes autonomy in the postpartum period. Women reported negative experiences when health professionals did not respect their preferences for childbirth or did not provide them with adequate information and support.
Attanasio et al.^8^ 2018 USA	*Women’s preference for vaginal birth after a first delivery by cesarean*	616 women with previous C-section, not pregnant and without a child within 12 months of C-section	Retrospective observational cohort study	Women’s previous birth experiences influenced their subsequent birth mode. A positive or negative experience led to repeating or avoiding previous experience. Women who wanted a VBAC wanted to avoid surgery, have an easier recovery, and wanted a larger family. Many women found it difficult to access VBAC support but the most frequent reason for a VBAC was the desire to experience vaginal birth.
Basile et al.^19^ 2020 USA	*Women’s Perceptions of Barriers and Facilitators to Vaginal Birth After Cesarean in the United States: An Integrative Review*	Women with previous experience of C-section or VBAC	Integrative review	Psychological factors: fear, loss of control, and risk concerns influenced decision-making. Empowerment was linked to knowledge about delivery options and recovery expectations. Social factors: family, friends, and cultural attitudes towards vaginal birth were key sources of support. Health factors: negative clinician language, limited insurance, and financial barriers were obstacles, while supportive hospital policies facilitated VBAC access.
Chen et al.^20^ 2017 Taiwan	*Influences on vaginal birth after caesarean section: A qualitative study of Taiwanese women*	29 women, with a previous C-section, pregnant, g.a. 34–38 weeks, eligible for a VBAC	Qualitative study	Negative experiences with C-sections: pain, discomfort, and difficulties with infant interaction, especially breastfeeding. Perceived benefits of C-sections: seen as quicker and more predictable, often chosen for perceived safety. VBAC concerns: fear of complications and influence of healthcare professionals towards ERCS. Cultural and partner influence: cultural factors and partner involvement also played a significant role in decision-making.
Chen et al.^9^ 2018 Taiwan	*Women’s decision-making processes and the influences on their mode of birth following a previous caesarean section in Taiwan: a qualitative study*	21 women with a previous C-section, low-risk pregnancy, g.a. 30–32 weeks, and in after birth	Grounded Theory study	Women’s main concerns were the health and wellbeing of both mother and baby. Some feared uterine rupture and labor pain, leading them to prefer repeat C-sections, while others believed vaginal delivery was better for health outcomes. External influences: experiences of family and friends, online information, and obstetrician recommendations influenced decision-making.
Davis et al.^5^ 2020 Australia	*Choosing vaginal birth after caesarean section: Motivating factors*	18 women with a previous C-section, low-risk pregnancy and eligible for a VBAC	Qualitative study	Women committed to natural birth, using their previous C-section experience to highlight recovery challenges. Their decision-making process involved customizing information to balance risks. Healthcare providers, especially midwives, played a crucial role in motivating women to choose vaginal delivery.
Fumagalli et al.^22^ 2020 Italy	*Women’s decision-making about mode of birth after a previous caesarean section*	76 women with a previous C-section, g.a. 34 weeks	Crosssectional study	Negative experiences from previous C-sections, such as feelings of body failure and loss of control, motivated women to attempt VBAC, seeking both physical and emotional healing. Despite obstetricians recommending C-sections, many chose VBAC for their perceived safety for the newborn, often seeking information online and from others with similar experiences, reflecting a lack of confidence in medical advice.
Hamilton et al.^11^ 2023 Australia	*Factors that Influence Women’s Decision on the Mode of Birth After a Previous Caesarean Section: A Meta-ethnography*	Women with previous experience of C-section or VBAC	Meta-ethnographic	Birth trauma, especially after emergency C-sections, negatively affects infant interaction, delivery satisfaction, and self-esteem, leading women to fear future birth and prefer C-sections. Health professionals’ support and practices such as encouragement and continuity of care are crucial for enhancing confidence in vaginal birth. Decisions about birth methods are often made early, and evidence-based education in the first trimester can promote informed choices. While partners influence decision-making, their role is acknowledged but not widely studied.
Konheim-Kalkstein et al.^18^ 2017 USA	*Owning the birth experience: what factors influence women’s vaginal birth after caesarean decision?*	173 pregnant women g.a.<35 weeks, with a single previous C-section and eligible for VBAC	Prospective observational cohort study	The desire to experience vaginal birth is the strongest predictor of VBAC choice. Women who want a VBAC are wanting to experience vaginal birth and feel it is natural. They have a desire to control their own body and felt it was safer for the baby. Women wanting a C-section wanted a controlled environment and a planned birth.
Lennon et al.^31^ 2023 Ireland	*VBAC or elective CS? An exploration of decision-making process employed by women on their mode of birth following a previous lower segment caesarean section*	13 women with a previous C-section and eligible for VBAC	Qualitative study	Views on delivery options and the role of support: some women see vaginal birth as a rite of passage into motherhood, but lack of postnatal discussions about VBAC leaves many unaware of this option. Some prefer C-sections for better control over timing and family planning. Fear of not achieving a VBAC and recovery experiences from previous C-sections can also influence their choice. Partners typically support women’s decisions, while insights from friends, family, and online communities play a significant role. Continuity of care is essential in the decision-making process.
Moysiadou^30^ 2023 Greece	*Vaginal birth after cesarean section: A quantitative study exploring women’s understanding and experience regarding VBAC rates in Greece*	454 women with a previous VBAC	Cross-sectional retrospective study	Women who had a successful VBAC reported more positive emotions and higher satisfaction than those who had a C-section. Key reasons for choosing VBAC include the desire for a vaginal birth, perceiving it as the natural way of giving birth, avoiding another surgery, and expecting a faster recovery. Partners provided more support than families in the decision-making process. Most women expressed positive emotions regarding the support and presence of healthcare professionals, especially midwives, during childbirth.
Munro et al.^10^ 2017 Canada	*Seeking control in the midst of uncertainty: Women’s experiences of choosing mode of birth after caesarean*	23 women with a previous C-section eligible for the VBAC	Grounded Theory study	Women may feel ‘failed’ if they are unable to achieve a vaginal delivery. Key factors influencing their decisions include the health of the newborn, immediate bonding, quick recovery, and respectful treatment from clinicians. Negative experiences, such as being separated from their newborn after a C-section, can affect interaction and breastfeeding. To fill knowledge gaps, women actively seek information, often turning to the internet and sharing birth stories. They prefer to discuss their priorities and receive information ahead of time to better prepare for childbirth.
Nilsson et al.^25^ 2017 Finland Netherlands Sweden	*Vaginal Birth After Cesarean: Views of Women From Countries With High VBAC Rates*	22 women with experience of VBAC	Qualitative study	Women seek personalized, early, and accurate information about VBAC, including potential complications. They value supportive healthcare professionals, a calm delivery environment, and continuity of care. Understanding the benefits of vaginal delivery can motivate them to opt for VBAC. Connecting with other women who have experienced VBAC is also beneficial. Fear remains a significant obstacle in the decision-making process. It is important for partners to share their birth experiences, as women prioritize the newborn’s safety and well-being above all.
Nilsson et al.^26^ 2017 Germany Ireland Italy	*Vaginal birth after caesarean: Views of women from countries with low VBAC rates.*	51 pregnant women with a previous C-section	Qualitative, descriptive study	Women want to be involved in decision-making, though the desired level of involvement varies. They seek timely information about VBAC, ideally right after a C-section, with a balanced view of the pros and cons of both VBAC and repeat C-sections. Having a personal birth plan and understanding available options during labor is also beneficial. Women suggest forming specific groups for VBAC support and meeting others with similar experiences.
Shorten et al.^17^ 2014 USA	*Complexities of Choice after Prior Cesarean: A Narrative Analysis*	187 women, g.a. 12–20 weeks, with a previous C-section eligible for VBAC	Qualitative study	The decision-making process regarding birth mode is shaped by multiple factors, with intense emotions such as fear and anxiety playing a central role. Women often prefer vaginal delivery or a repeat C-section based on past experiences, expectations of quicker recovery, and concerns about the newborn’s safety. Obstetricians’ recommendations strongly influence these choices, while access to clear and reliable information helps women feel more confident in their decisions.
Shurong et al.^21^ 2024 China	*Decision-making experiences and the need for decision aids in women considering vaginal birth after cesarean: A qualitative meta-synthesis*	Women who prefer VBAC or experienced it	Systematic review, metasynthesis	The decision-making process is influenced by various factors, including family pressures, inadequate support from healthcare professionals, and internal conflicts about the ability to give birth. Common psychological challenges such as fear, anxiety, and uncertainty are prevalent. Women often report a sense of loss of control during their previous delivery and view the subsequent birth as an opportunity to regain that control. They emphasize the importance of receiving detailed information, including statistics, and seek emotional support. Many women prefer obtaining information through advice and interactions with others who have had similar experiences.
Simeone et al.^23^ 2019 Italy	*Experience of Vaginal Birth After Cesarean: A Phenomenological Study*	11 women with previous successful VBAC within 6 weeks	Qualitative study	Women reported these main themes: description of previous experience, desire for naturalness, poor information, vivid memories of VBAC, the perception of assistance (positive perception of continuity presence of healthcare professionals), and psychological support received.
Sys et al.^28^ 2021 Poland	*Women’s views of birth after cesarean section*	Women with previous experience of C-section or VBAC	Narrative literature review	Preferences for birth mode after a C-section are influenced by cultural, social, and personal factors. Women who prefer VBAC typically have a better understanding of its risks and benefits compared to those opting for repeat C-sections. They are more likely to engage in childbirth preparation courses and actively seek information online and from peers. Support from clinicians, particularly midwives, plays a crucial role in the decision-making process. Women who have successful VBAC experiences report positive outcomes, while those opting for C-sections often do so to avoid the risks associated with emergency C-sections and for perceived safety.
Sys et al.^27^ 2022 Poland	*Women’s views and preferences regarding the mode of birth after cesarean section: Polish cross-sectional web-based survey*	733 women with previous C-section eligible for VBAC	Cross-sectional study	Most women preferred vaginal birth after a C-section, especially those with prior vaginal births, who were more likely to attempt VBAC, viewing childbirth as a physiological process. Their motivations included faster recovery, better health for both mother and newborn, skin-to-skin contact, and improved breastfeeding conditions. However, concerns about negative clinician attitudes and the possibility of TOLAC ending in a C-section influenced their decision-making. Women who preferred C-sections were more focused on risks and their rights. The Internet was a primary source of information, with women interested in VBAC engaging more by reading, preparing birth plans, sharing experiences, joining support groups, and attending childbirth preparation courses.
Triunfo et al.^24^ 2019 Italy	*Socio-cultural and clinician determinants in the maternal decision-making process in the choice for trial of labor vs. elective repeated cesarean section: a questionnaire comparison between Italian settings*	133 women who experience at least a previous C-section	Prospective observational cohort study	Women consider various factors when choosing their delivery method, including quick recovery, less pain, avoidance of episiotomy, the ability to schedule childbirth, and post-birth sexual quality. Support from healthcare providers and family is crucial for opting for a vaginal birth. Prenatal counseling, personalized assistance during delivery, and strong motivation are key to deciding on a VBAC. Past negative childbirth experiences may lead to opting for a C-section, but a failed labor does not necessarily deter the choice of VBAC.
Williams et al.^4^ 2021 USA	*Factors leading to satisfaction with counseling for Labor after Cesarean among Latina women in the United States*	11 pregnant women who had received counselling about VBAC	Qualitative study	Three key themes emerged regarding counseling on VBAC: factors affecting satisfaction, influences on decision-making, and preferences for the counseling method and timing. Women expressed higher satisfaction when clinicians communicated clearly and reliably, showing awareness of their experiences and facilitating informed choices. Their decisions were influenced by past birthing experiences, the desire for a safe delivery and quick recovery, and considerations for future family planning.

TOLAC: Trail of Labor After Cesarian.

### Data synthesis

Extracted data were systematically categorized and synthesized through thematic analysis, allowing for the identification of key themes and sub-themes. This qualitative approach enabled a comprehensive and structured interpretation of the findings, providing meaningful insights into the research questions^16^.

## RESULTS

A total of 492 articles were identified through the research strategy. After applying inclusion and exclusion criteria, 167 duplicates were removed, 291 studies were excluded based on title screening, 10 were excluded after Abstract review, and 4 were excluded following full-text analysis. Of the four studies excluded after full-text screening, all were removed because they did not meet the inclusion criteria. Specifically, they focused exclusively on clinical outcomes related to maternal or neonatal health, without addressing women’s lived experiences, perceptions, or decision-making regarding VBAC. As a result, 21 studies were included in the final review (Figure 1).

No studies were excluded based on the Critical Appraisal Skills Program (CASP) criteria^13^. A summary of the selected studies is provided in Table 1. Of the 21 included studies, 5 were conducted in the United States^4,8,17-19^, 2 in Australia^5,11^, 2 in Taiwan^9,20^, 1 in Canada^10^, 1 in China^21^, 3 in Italy^22-24^ and 1 in Finland, the Netherlands and Iceland^25^, 1 in Germany, Ireland and Italy^26^, 2 in Poland^27,28^, 1 in Turkey^29^, 1 in Greece^30^, and 1 in Ireland^31^.

More than half of the studies (n=11/21) were qualitative and within these, various research approaches were employed: 5 studies utilized a descriptive approach^5,20,25,26,31^, 2 adopted a phenomenological approach^23,29^, 2 applied Grounded Theory^9,10^, 1 used an inductive approach^4^ and 1 employed narrative analysis^17^.

Four of the included articles were literature reviews: one systematic with a qualitative meta-synthesis approach^21^, one meta-ethnographic approach^11^, one narrative review^28^ and one integrative review^19^. Three studies were cross-sectional observational studies^22,27,30^, while the remaining three were cohort studies, comprising 2 prospective studies^18,24^ and one retrospective study^8^.

**Figure 2:**
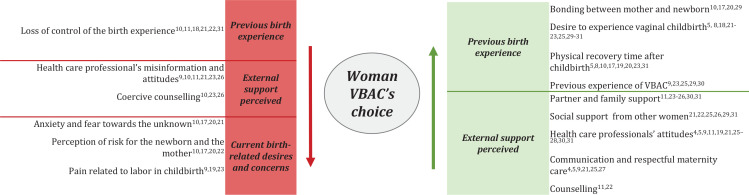
Facilitators and barriers to the choice of VBAC

The systematic review identified 12 key factors that women perceived as influencing their decision-making process regarding the mode of birth in subsequent pregnancies following a previous CS, henceforth referred to as ‘factors’. These include: bonding between mother and newborn; desire for a vaginal birth; physical recovery time after childbirth; previous experience of VBAC; partner and family support; social support from other women; attitudes, skills and support of healthcare professionals; loss of control over the birth experience; anxiety and fear of the unknown; perception of risk for the newborn and the mother; pain associated with labor and childbirth and unmet information needs (Figure 2). These factors were categorized as either ‘facilitators’ or ‘barriers’ to the choice of VBAC, depending on their influence on maternal choice. Facilitators and barriers were framed into ‘Previous birth experience’, ‘External support perceived’ and ‘Current birth-related desires and concerns’ (Figure 2).

### Facilitators

*Previous birth experience*

Bonding between mother and newborn

The importance of early bonding between mother and newborn emerged as one of the most frequently cited factors among women with a previous C-section. Research consistently showed that the experience of a previous C-section negatively affected women’s physical, psychological and social health, particularly in terms of mother–infant interaction during the immediate postpartum period^10,17,20,29^. Women often expressed regret over the missed opportunity for bonding and breastfeeding^17^ and reported a profound loss of maternal identity. Many cited the immediate separation from their newborn as a key negative aspect of their previous birth experience. Consequently, uninterrupted skin-to-skin contact was defined as a crucial factor for subsequent VBAC decisions, with many women seeking VBAC in the hope of achieving better health outcomes for their newborn and initiation breastfeeding^10,20^.

Desire to experience vaginal childbirth

Many women with a history of CS viewed vaginal birth as a normal, natural and safe option, which they believed would improve bonding between mother and newborn^5,8,23,30^. The desire was often linked to a sense of loss over missing out on the experience of natural birth^18,25^. For some, vaginal birth was seen as culturally sanctioned, while others sought to avoid unnecessary surgery^5,21^. VBAC was viewed as a rite of passage and an essential part of motherhood and femininity^29,31^.

Conversely, C-sections were often perceived as a failure in the journey to motherhood^29,31^. In line with this, Fumagalli et al.^22^ found that the maternal desire to achieve vaginal birth and feelings of regret over the previous C-section were independent predictors of VBAC.

Physical recovery time after childbirth

Concerns related to post-surgical recovery were prevalent among women with previous C-sections. Many women expressed a desire for a VBAC in the hope of quicker recovery and the ability to care for their families more effectively^5,19,20^. Women who had experienced difficult postcesarean recoveries, particularly those lacking strong social or family support, reported that VBAC would allow for a quicker return to autonomy and caregiving responsibilities^10^. Additionally, women who successfully achieved VBAC reported greater freedom and readiness to care for their newborns, both physically and mentally^23^. However, some studies found that while post-surgical recovery could influence the decision, it was not a definitive predictor^8,10,17^. Women who had a positive C-section recovery tended to opt for another C-section, while those with more complicated recoveries were more inclined to pursue VBAC^31^.

Previous experience of VBAC

Women with prior VBAC experience often reported mixed emotions. While VBAC offered a sense of ‘revenge’ against the loss of control during the previous C-section, it was also described as a ‘healing’ experience. Despite these positive feelings, many women expressed fear of the pain associated with vaginal birth^23,29^. Interestingly, the pain of the trial of labor was often compared to the pain of a previous C-section, with women describing labor pain as intense but ultimately transient and essential^9,23^.

For some, sharing a calm, familiar VBAC experience with support from loved ones led to increased happiness and a stronger sense of care during labor^23,25,29,30^.

A notable finding from Moysiadou^30^ is that women who previously had VBAC would often choose it again for future pregnancies.

*External support perceived*

Partner and family support

Although the role of partners in the decision-making process regarding VBAC is still understudied, existing research suggests that they play a significant role in influencing a woman’s choice of mode of birth^11^. Several studies have highlighted the supportive role of partners^11,26,30,31^. This support was particularly evident in women with low self-confidence or those who lacked shared decision-making opportunities within a multidisciplinary team. In such cases, the partner’s presence helped alleviate the burden of choice and facilitated alignment between the women’s preferences and those of healthcare providers^11,25^.

Studies have also described varying roles of partners in the decision-making process. While many act as supporters, some function as deterrents. In certain cases, a partner’s negative birth experience influenced their own perception of birth options, leading them to prefer an elective C-section to avoid the unpredictability associated with a VBAC^26,31^.

Family members also played a role in shaping women’s decision-making. Their involvement was found to enhance compliance and confidence in VBAC decisions^24,25,31^. However, some studies reported a lack of family support^23,25^, which negatively impacted a woman’s autonomy in choosing VBAC. In some cases, women only received support after overcoming initial resistance related to cultural preconceptions which often contributed to social prejudices that limited their ability to make independent birth choices^23^.

Social support from other women

The experiences shared by other women who had previously undergone childbirth play a crucial role in decision-making, particularly in mitigating negative emotions and fears associated with VBAC^21,31^.

Hearing first-hand birth stories and accessing online information were key influencing factors in the decision to pursue VBAC^22^. Women often sought support from multiple sources, including conversations with friends, social media platforms, online forums, educational training, Internet research, academic articles and books^25,31^.

Online support groups were particularly valuable, offering a safe space for women to engage in discussion, exchange experiences and receive encouragement about their VBAC choices^21^. This form of peer support helped women feel enlightened, empowered and motivated in their decision-making process^29^. Women highlighted several benefits of these groups, including emotional validation, realistic birth information and opportunities to process previous birth experiences^25,26^.

Healthcare professionals’ attitudes

The attitudes, skills and level of support provided by healthcare professionals significantly influence women’s decisions regarding VBAC. Care pathways, access to information and the approach taken by healthcare providers play a central role in shaping maternal choices. All studies that examine this factor indicate that healthcare professionals’ opinions and knowledge affect women’s preferences between attempting a VBAC or opting for a repeat C-section^4,5,9,11,19,21,25-28,30,31^.

Among healthcare professionals involved in the decision-making process, midwives were more likely to support VBAC compared to obstetricians^5,27^. Additionally, women who received continuity of care from a midwife were more likely to choose and achieve a VBAC than those who received continuity of care from obstetricians^31^.

Communication and respectful maternity care

Women emphasized the importance of feeling supported by healthcare professionals throughout the decision-making process, whether they were doctors or midwives. They highlighted the need for autonomy in decision-making, based on trust in the accuracy and objectivity of the information provided^4,5,9,21,25,27^.

Moreover, active listening was a key expectation from healthcare professionals. Women reported that respectful and individualized care helped them feel more confident in their choices^25^. Alongside clinical expertise, women expected compassion, empathy, patience, and acknowledgment of their fears from their healthcare providers.

Counseling

A positive and encouraging approach to vaginal birth emphasizing its benefits rather than focusing solely on potential risks was associated with higher rates of VBAC uptake^11^. Finally, the perceived risk associated with repeat C-sections was a key determinant in women’s decision-making. Studies found a statistically significant association between women perceiving multiple C-sections as high-risk procedures and their increased likelihood of choosing VBAC^22^. Women who believed that undergoing multiple C-sections posed greater health risks were more likely to opt for VBAC.

### Barriers

*Previous birth experience*

Loss of control of the birth experience

The feeling of loss of control during a previous childbirth experience, particularly when associated with low maternal satisfaction, has been identified as a significant barrier to choosing VBAC^22^. Women who underwent an unplanned C-section during labor often reported experiencing a sense of powerlessness during birth. As a result, many opted for an elective repeat C-section in subsequent pregnancies, viewing it as a more predictable and controlled mode of birth^10,11,21,31^.

Additionally, some women feared that they might not be able to achieve a ‘successful’ VBAC, as it was an unknown and uncertain experience for them^31^. The perception of losing control during previous birth was found to be negatively associated with the choice of VBAC^18^.

*External support perceived*

Women often reported dissatisfaction with the information they received and the lack of adequate support from healthcare professionals regarding VBAC^21^.

Health care professional’s misinformation and attitudes

Women frequently felt that the answers they received were ‘inappropriate’ and ‘insistent’ and were rarely complete^21^. This contributed to an increased feeling of lack of support, education and preparation needed for the decision-making process regarding the mode of birth^9,10,26^.

Women expressed a need to be supported by healthcare professionals who provide consistent information about VBAC. Specifically, they reported feelings of having their concerns minimized^26^ and noticed significant differences in the content of information due to personal healthcare professionals’ attitudes and preferences (VBAC vs repeat C-section)^9^. Moreover, women reported a lack of healthcare professionals’ knowledge regarding VBAC, leading to inadequate information about the risks and benefits of vaginal birth after a C-section, especially when interpreting statistical data^9,10,26^. Some women were even unaware that VBAC was an option after a C-section^26^.

In response to the lack of adequate information regarding the VBAC theme, women often turned to personal research to inform themselves, utilizing sources such as the Internet, personal knowledge, scientific literature, blogs and social media^9,11,21,23^.

Coercive counseling

Several studies reported that healthcare professionals used ‘intimidating tactics’ to promote repeated C-sections, including threats of death for both the mother and the newborn due to complications (such as uterine rupture, postpartum hemorrhage, or hysterectomy), which increased women’s fears about the risks of vaginal labor and birth after a previous C-section^10,23,26^.

*Current birth-related desires and concerns*

Anxiety and fear of the unknown

Anxiety and fear regarding the uncertainty of vaginal birth were among the most commonly reported psychological barriers to VBAC. These emotions significantly reduced women’s confidence in attempting a vaginal birth after a C-section. Such fear often stemmed from: past negative birth experiences, concerns about labor pain, potential complications for both mother and infant, influence of family members’ opinions, medical recommendations and inconsistent information. These factors collectively contributed to some women perceiving a repeat C-section as a safer option^17,20,21^.

Munro et al.^10^ further noted that unplanned C-sections were more likely to lead to postpartum anxiety and depression. Consequently, planning a repeat elective C-section became a strategy for some women to avoid the unpredictability of labor and to repeat a familiar birth experience^10^.

Perception of risk for the newborn and the mother

Research has shown that maternal choice of mode of birth is strongly associated with the perceived risk for the newborn’s health^22^. While many women acknowledged the potential benefits of vaginal delivery, their fear of neonatal complications often led them to choose a repeat C-section^17^.

The primary concern underlying women’s hesitation towards VBAC was the perceived risk of uterine rupture, which could endanger the baby’s health. These findings suggest that women prioritize neonatal safety above other considerations, influencing them to opt for a repeat C-section despite its own risks^10,17,20^.

Pain related to labor and childbirth

Pain during labor has been identified as one of the most significant deterrents to VBAC decision-making^9^. Labor pain, recognized as both a sensory and emotional experience, represents a considerable barrier for women considering VBAC after a previous C-section^19^. Women who had negative experiences with labor or ‘failed’ vaginal births often expressed fears about their ability to cope with the pain. Many perceived their bodies as inadequate for vaginal birth, fearing both labor pain and the possibility of postoperative pain in the event of another emergency C-section which sometimes led them to opt for a scheduled repeat C-section instead^19,23^.

## DISCUSSION

This systematic review examines the key factors influencing women’s choice of birth mode in subsequent pregnancies following a previous C-section, identifying both facilitators and barriers (Figure 2). Our findings synthesize these influences into 12 distinct factors spanning psychological, biological, social, and healthcare domains. Of these, 7 factors functioned as facilitators, while 5 acted as barriers, shaping decision-making regarding the mode of birth after a prior C-section.

Our findings confirm that women’s decision-making process regarding VBAC is shaped by multiple interrelated factors, including their previous birth experience, the external support they perceived, and their current birth-related desires and concerns. Regarding their previous experience, our study identifies four key facilitators: the bonding between mother and newborn, the desire for a vaginal birth, the shorter physical recovery time after childbirth, and a previous successful VBAC. Conversely, one barrier is the sense of loss of control over the previous birth experience, which may lead to apprehension about attempting a vaginal birth after cesarean. When considering external support, research highlights three main facilitators: the presence of strong support from a partner, family, social support, and other women, as well as the attitudes, communication skills, and counseling provided by healthcare professionals. However, critical barriers in this domain are the healthcare professional’s misinformation and attitudes and coercive counseling, which may leave women feeling uncertain or inadequately prepared to make an informed decision. Finally, regarding their actual birth experience and desire, the literature identifies three principal barriers that may discourage women from choosing VBAC: anxiety and fear of the unknown, perceived risks for both the newborn and the mother, and the pain associated with labor and childbirth. Women’s previous experience and external support contain all the facilitators identified by our systematic revision, underlining their importance in promoting VBAC choice.

Within all the factors described, the role of healthcare professionals, described in the care factors, emerged as one of the most frequently discussed in the included studies (Figure 2). The attitude and knowledge of healthcare professionals can act as a facilitator when they endorse VBAC as a safe option, providing individualized care and promoting shared decision-making^11,25^. However, these factors become barriers when women experience insufficient information and lack of support, increasing fears regarding the risks of vaginal birth after C-section^10,23,32^. Women emphasized the need for clear, accurate, reliable and accessible information, including statistical data on VBAC success rates^9,10,32^.

Counseling during the decision-making process emerged as a crucial factor in both facilitating and hindering VBAC choices^4,33^. Effective counseling, which provides unbiased and balanced information, can empower women and place them at the center of decision-making. This approach should reinforce facilitators and mitigate barriers, while promoting support from family, social networks, and peers^33^.

Midwives were highlighted as the most supportive healthcare professionals in facilitating positive VBAC experiences^5,27^. Their expertise in providing information and education ensures women feel confident and informed in making their birth choices^21^. Midwives also promote a proactive role in decision-making, addressing fear and anxiety about hildbirth^10,21^. Women value healthcare professionals who listen carefully, provide individualized support, and offer a respectful and optimistic approach to VBAC^11,25^. These non-clinical aspects, including communication and respectful care, are essential to improving maternal satisfaction and increasing VBAC uptake^34^. In this context, continuity models in maternity care have been linked to improved VBAC experience. These models are associated with higher VBAC attempt rates, greater VBAC success rates, and a higher likelihood of spontaneous labor^35^. A Cochrane review further supports that midwifery continuity models enhance perinatal outcomes, improve birth experiences, and reduce healthcare costss^36^.

### Strength and limitations

The primary strength of this study lies in the rigorous methodology used to search, select and synthesize existing literature. The review followed PRISMA guidelines, included multiple databases, and employed quality appraisal tools to ensure methodological rigor.

However, some limitations must be acknowledged. First, the language restriction to English-only publications may have led to the exclusion of relevant studies conducted in other languages. Second, the geographical distribution of the included studies is heavily skewed toward high-income countries, such as the USA, UK, Australia, and European contexts. This limits the cultural and healthcare system diversity represented in the synthesis and may reduce the applicability of the findings to low- and middle-income countries, where access to VBAC and maternity care models can differ substantially.

## CONCLUSIONS

The findings of this review underscore the complex nature of VBAC decision-making, influenced by psychological, social, biological, and care factors. Women’s choices are shaped by their previous birth experiences, the external support they perceive, and their perceptions of risk and benefit concerning the actual birth. Addressing these factors through high-quality, unbiased counseling and individualized support is crucial for fostering informed and autonomous decision-making. Improving healthcare professionals’ training and developing standardized protocols for VBAC counseling could significantly influence women’s perceptions and help overcome medical biases against VBAC.

Healthcare professionals play a pivotal role in this process. Their attitudes, knowledge, and communication skills can greatly impact women’s confidence in VBAC. Midwives, in particular, have been recognized as key facilitators in promoting positive VBAC experiences. Their ability to offer continuity of care, emotional support, and comprehensive education contributes to improved maternal satisfaction and higher VBAC success rates.

Finally, continuity models of maternity care, especially those led by midwives, have been linked to better birth outcomes, increased VBAC rates, and reduced healthcare costs. Therefore, expanding continuity of care and integrating early multidisciplinary prenatal counseling could empower women to make informed birth choices. The development of midwife-led prenatal education programs for women with previous C-sections – incorporating peer discussions and professional guidance – could reduce uncertainty and increase confidence in VBAC^21,22,31^.

Future research should focus on developing decision-support tools to aid VBAC counseling, utilizing evidence-based information and risk assessments to empower women in their choices. Additionally, training programs for healthcare professionals should be enhanced to improve VBAC counseling, with an emphasis on shared decision-making and effective communication.

## Data Availability

The search strategy and data extraction form are available from the corresponding author upon reasonable request.
